# Olive Leaf Extract, from *Olea europaea* L., Reduces Palmitate-Induced Inflammation via Regulation of Murine Macrophages Polarization

**DOI:** 10.3390/nu12123663

**Published:** 2020-11-28

**Authors:** Paola De Cicco, Maria Maisto, Gian Carlo Tenore, Angela Ianaro

**Affiliations:** Department of Pharmacy, School of Medicine, University of Naples Federico II, 80138 Naples, Italy; maria.maisto@unina.it (M.M.); giancarlo.tenore@unina.it (G.C.T.); ianaro@unina.it (A.I.)

**Keywords:** obesity, nutraceuticals, olive leaf, macrophages, inflammation

## Abstract

Olive tree (*Olea europaea* L.) leaves are an abundant source of bioactive compounds with several beneficial effects for human health. Recently, the effect of olive leaf extract in obesity has been studied. However, the molecular mechanism in preventing obesity-related inflammation has not been elucidated. Obesity is a state of chronic low-grade inflammation and is associated with an increase of pro-inflammatory M1 macrophages infiltration in the adipose tissue. In the current study, we explored *Olea europaea* L. leaf extract (OLE) anti-inflammatory activity using an in vitro model of obesity-induced inflammation obtained by stimulating murine macrophages RAW 264.7 with high dose of the free fatty acid palmitate. We found that OLE significantly suppressed the induction of pro-inflammatory mediators, tumor necrosis factor (TNF)-α, interleukin (IL)-6, IL-1β, nitric oxide (NO), prostaglandin E2 (PGE2) and reactive oxygen species (ROS), while it enhanced the anti-inflammatory cytokine, IL-10. Moreover, we demonstrated that OLE reduced the oxidative stress induced by palmitate in macrophages by regulating the NF-E2-related factor 2 (NRF2)−Kelch-like ECH-associated protein 1 (KEAP1) pathway. Finally, we showed that OLE promoted the shift of M1 macrophage toward less inflammatory M2-cells via the modulation of the associated NF-κB and proliferator-activated receptor gamma (PPARγ) signaling pathways. Thereby, our findings shed light on the potential therapeutic feature of OLE in recovering obesity-associated inflammation via regulating M1/M2 status.

## 1. Introduction

Obesity is one of the main threats to global human health and life expectancy. According to the World Health Organization (WHO), obesity levels have nearly tripled since 1975 and it has been estimated that in 2016 about 13% of the world’s adult population (11% of men and 15% of women) were obese [1]. High-fat diet (HFD)-induced obesity is associated with a chronic state of low-grade inflammation which increases the risk of developing obesity-associated diseases such as type 2 diabetes, cardiovascular diseases, musculoskeletal disorders and cancer [2]. The major players implicated in the inflammatory response observed in the obese adipose tissue are the pro-inflammatory macrophages [3,4,5]. Macrophages are a heterogeneous population of cells that are instrumental in initiating the innate immune response. They represent almost the 40% of all adipose tissue cells in obese mice compared to 10% in lean mice [3]. In addition to their numbers, adipose tissue macrophages (ATMs) in lean and obese animals exhibit distinct phenotype and functions. In particular, two different macrophage populations have been found in adipose tissue: the classically activated macrophages or M1 (pro-inflammatory) and the alternatively activated macrophages or M2 (anti-inflammatory). In the lean state, M2 macrophages predominate in order to maintain tissue homeostasis and insulin sensitivity by secreting the anti-inflammatory cytokine interleukin (IL)-10. By contrast, during weight gain, M1 macrophages proliferate greatly and replace the M2 macrophages to control and sustain a chronic inflammation state through the release of pro-inflammatory mediators, such as tumor necrosis factor (TNF)-α, IL-6, IL-1β, reactive oxygen species (ROS), prostaglandin E2 (PGE2) and nitric oxide (NO) [6,7]. ATM numbers and/or pro-inflammatory gene expression are negatively associated with weight loss in obese subjects [8]. Moreover, the ratio of M1 to M2 cells in adipose tissue are positively correlated to the incidence of insulin resistance in obesity [9]. Diet and lifestyle may affect ATM polarization in obesity. Indeed, a saturated fatty acid (SFA)-rich diet induced upregulation of inflammatory genes and chemokines expression. Similarly, a high-carbohydrate meal also promotes inflammatory activation of macrophages [10,11]. Obese patients have significantly increased levels of free fatty acids (FFAs), in particular palmitate, in the blood [12]. Palmitate has been postulated to induce an inflammatory response mediating classical activation of macrophages in obesity by directly engaging toll-like receptors (TLR) and inducing Nuclear Factor kappa-light-chain-enhancer of activated B cells (NF-κB)-dependent production of inflammatory cytokines such as TNF-α and IL-6 and by generation of ROS [13,14]. Accordingly, manipulation of M1/M2 homeostasis has been shown to be an effective strategy to control obesity and obesity-related diseases. Indeed, recent studies have demonstrated that infusion of IL-4, which is necessary for M2 macrophage activation, or M2 macrophages themselves could ameliorate obesity and insulin resistance in HFD mice [15,16]. Natural products provide abundant resources for anti-inflammatory compounds with potential benefits for obese patients. Various dietary components including long chain omega-3 fatty acids, antioxidant vitamins, plant flavonoids, prebiotics and probiotics have demonstrated the potential to prevent chronic inflammatory conditions [17]. *Olea europaea* L. is a fruit tree native to Asia Minor and Syria, which is now widely cultivated in the Mediterranean region. The main product extracted from the olive tree is extra-virgin olive oil (EVOO), one of the bases of the Mediterranean Diet, which is very popular for its nutritive and healthy effects particularly given the high contents of monounsaturated fatty acids as well as of other minor and worthy components like phenolics, phytosterols, tocopherols and squalene (1–2%) [18]. However, olea by-products represent rich sources of bioactive molecules; in particular, olive leaves contain phenolic compounds in amounts higher than EVOO. For example, the amount of oleuropein, which is the most abundant phenolic compound in olive leaves, ranges from 0.005% and 0.12% in EVOO while in olive leaves it ranges between 1% and 14% [19]. Among the other main polyphenols identified in olive leaf extract are hydroxytyrosol, verbascoside, apigenin and luteolin [20]. Over the centuries, extracts from olive leaves have been used for the treatment of many diseases thanks to their numerous beneficial effects to human health, such as anti-oxidant, anti-hypertensive, cardioprotective and anti-inflammatory effects [18]. Moreover, olive leaf extract has demonstrated anti-obesity effects by regulating molecular pathways involved in thermogenesis and adipogenesis [21,22]. However, the molecular mechanism of olive leaf extract in preventing obesity-related inflammation has not been elucidated. Thus, in the present study, we investigated the anti-inflammatory effects of *Olea europaea* L. leaf extract using an in vitro model of obesity-induced inflammation obtained by stimulating murine macrophages RAW 264.7 with a high dose of palmitate. Furthermore, we report novel findings relating to the ability of OLE to regulate the M1/M2 status via the modulation of the associated NF-κB and proliferator-activated receptor gamma (PPARγ) signaling pathways.

## 2. Materials and Methods

### 2.1. Cell Culture

RAW 264.7 macrophage cells were purchased from the American Type Culture Collection (ATCC), and cultured in Dulbecco’s Modified Eagle Medium (DMEM) containing 10% fetal bovine serum (FBS), 2 mmol/L L-glutamine, penicillin (100 U/mL), streptomycin (100 µg/mL) and 1 mmol/L sodium pyruvate (all from Gibco, Thermo Fisher Scientific, Rodano (MI), Italy). Cells were grown at 37 °C in a humidified incubator under 5% CO_2_.

### 2.2. Sodium Palmitate (SP) Preparation

Sodium palmitate (P9767; Sigma-Aldrich) was prepared by diluting a 200 mM stock solution in 70% ethanol into 10% fatty acid–free, low-endotoxin bovin serum albumin (BSA) (A-8806; Sigma-Aldrich; adjusted to pH 7.4) by heating at 50 °C. The palmitate-BSA stock solution was filtered using a 0.22-µm low-protein binding filter (Millipore, Billerica, MA, USA). Sodium palmitate was added at 0.5 mM. BSA/70% ethanol (10%) was used as vehicle in control cells.

### 2.3. Olive Leaf Extract (OLE) Preparation

Olive leaf extract was obtained by Olea europea L cultivar Ravece. Fresh leaves were collected from the rural region of Avellino, in the south of Italy, in the month of October 2019. 10 g of lyophilized raw material were extracted with 100 mL pure ethanol (1.59010; Sigma; food-grade solvent) for 4 h in darkness and at room temperature. The extract obtained was centrifuged (10 min at 8000 rpm) and supernatant underwent a spray-drying process with maltodextrins (Farmalabor, Canosa, Italy) as support, obtaining a fine powder, which was used for the in vitro experiments. This product was formulated by the Department of Pharmacy, University of Naples “Federico II” (Naples, Italy). Large scale production of OLE has been accomplished by MB Med Company (Turin, Italy).

### 2.4. High-Performance Liquid Chromatography (HPLC)/Diode-Aray Detector (DAD) Analyses of OLE Polyphenols

The content of polyphenols in OLE were monitored by HPLC-DAD analysis, following the method described by Xie et al., with slight modifications. Analyses were run on a Jasco Extrema LC-4000 system (Jasco Inc., Easton, MD) provided with photo DAD. The column selected was a Kinetex^®^ C18 column (250 mm × 4.6 mm, 5 μm; Phenomenex, Torrance, CA, USA). The analyses were performed at a flow rate of 1 mL/min, with solvent A (0.1% formic acid) and solvent B (0.1% formic acid in acetonitrile) and monitored at the absorbance of 280 nm, 338 nm and 360 nm. The elution gradient was performed according to the following conditions: from 15% (B) to 40% (B) in 20 min, to 95% (B) in 10 min and to 15% (B) in 2 min, followed by 8 min of maintenance. For quantitative analysis, standard curves for each polyphenol standard were prepared over a concentration range of 0.1–1.0 μg/μL with six different concentration levels and duplicate injections at each level.

### 2.5. Quantitative Real-Time PCR

RAW 264.7 cells (1 × 10^6^ cells/well) or Bone Marrow Derived Microphages (BMDMs) (1 × 10^6^ cells/well) were treated with OLE (0.1 and 0.2 mg/mL) for 30 min before stimulation with SP (0.5 mM) for 6 h or 24 h. In the experiments with GW9662, cells were pre-incubate with GW9662 (Sigma-Aldrich, Milan, Italy) 1 µM for 24 h. Total RNA was extracted from macrophages by using TRI-Reagent (Sigma-Aldrich, Milan, Italy), according to the manufacturer’s instructions, followed by reverse-transcription with iScript Reverse Transcription Supermix for RT-qPCR (Bio-Rad, Milan, Italy). Quantitative Real-Time PCR (RT-PCR) was performed by using CFX384 real-time PCR detection system (Bio-Rad, Milan, Italy). mRNA expression was quantified using specific primers for mouse *Il-6, Il-1β, Tnf-α, Il-10, iNos (Nos2), Cox-2 (Ptgs2), Pparγ, Cd206, Arg-1, Gclc, Gclm, Hmox-1*, which are listed below, with SYBR Green master mix kit (Bio-Rad, Milan, Italy). Relative gene expression was obtained by normalizing the Ct values of each experimental group against *β-actin* transcript level, using the 2-ΔCt formula. mRNA levels are expressed as arbitrary units (A.U.)

*Il-6*: 5′-CGGAGAGGAGACTTCACAGAG-3′; 5′-ATTTCCACGATTTCCCAGAG-3′

*Il-1β*: 5′-TACCAGTTGGGGAACTCTGC-3′; 5′-GGGCCTCAAAGGAAAGAATC-3′

*Tnf-α*: 5′-CAGTAGACAGAAGAGCGTGGT-3′; 5′-AGGCACTCCCCCAAAAGA-3′

*Il-10*: 5′-CGGAAACAACTCCTTGGAAA-3′; 5′-AAGTGTGGCCAGCCTTAGAA-3′

*Hmox-1*: 5′-GCCGTGTAGATATGGTACAAGGA-3′; 5′-AAGCCGAGAATGCTGAGTTCA-3′

*Gclm*: 5′-AGGAGCTTCGGGACTGTATCC-3′; 5′-GGGACATGGTGCATTCCAAAA-3′

*Gclc*: 5′GTTGGGGTTTGTCCTCTCCC-3′; 5′-GGGGTGACGAGGTGGAGTA-3′

*Arg-1*: 5′-CTGGTTGTCAGGGGAGTGTT-3′; GTGAAGAACCCACGGTCTGT-3′

*iNos*: 5′-CGAAACGCTTCACTTCCAA-3′; 5′-TGAGCCTATATTGCTGTGGCT-3′

*Cd206*: 5′- AGGACATGCCAGGGTCACCTTT-3′; 5′-GTTCACCTGGAGTGATGGTTCTC-3′

*Cox-2*: 5′-CCTGCTTGAGTATGTCGCAC-3′; 5′-TACCCTCCTCACATCCCTGA-3′

*Pparγ*: 5′-GTACTGTCGGTTTCAGAAGTGCC-3′; 5′-ATCTCCGCCAACAGCTTCTCCT-3′

*β-actin*: 5′-TACCACCATGTACCCAGGCA-3′; 5′-CTCAGGAGGAGCAATGATCTTGA-3′

### 2.6. Intracellular ROS Measurement

The generation of ROS was estimated using the fluorescence probe 2′,7′-dichlorofluorescein-diacetate (H2DCF-DA). RAW 264.7 macrophage (3 × 10^3^ cells/well) were plated in 96-multiwell black plates (Corning, USA) and incubated with OLE (0.1 and 0.2 mg/mL) for 30 min before to be stimulate with SP (0.5 mM) for 24 h. At the end of the treatment, cells were incubated with H2DCF-DA (100 μM) for 1 h. Then, cells were washed with PBS and incubated with the Fenton’s reagent (H_2_O_2_/Fe^2+^ 2 mM) for 3 h at 37 °C. Fluorescence generation was measured using a fluorescent microplate reader (excitation 485 nm and emission 538 nm; GloMax^®^-Multi Detection System, Promega). The intracellular ROS levels were expressed as fluorescence intensity.

### 2.7. Cytokines Measurement

The levels of cytokines (IL-6, IL-1β, TNF-α and IL-10) were evaluated in cell culture supernatants obtained from RAW 264.7 cells treated with OLE (0.1 and 0.2 mg/mL) for 30 min before stimulation with SP (0.5 mM) for 24 h. Enzyme-linked immunosorbent assay (ELISA) kits were used according to the manufacturer’s instructions (DuoSet ELISA, R&D systems, Minneapolis, MN, USA).

### 2.8. Nitrite Analysis

RAW 264.7 cells (2 × 10^5^ cells/well) were treated with OLE (0.1 and 0.2 mg/mL) for 30 min before stimulation with SP (0.5 mM) for 24 h. After stimulation, NO levels were spectrophotometrically evaluated in culture supernatant according to the Griess reaction method. Briefly, 100 µL of Griess reagent (0.1% naphthyl-ethylene-diamine dihydrochloride in water and 1% sulphanilamide in 5% concentrated H_3_PO_4_; vol. 1:1) was mixed with culture supernatant (100 µL). Absorbance was measured with a microplate spectrophotometer (MultiskanTM GO Microplate Spectrophotometer) at 540 nm and nitrite concentration was calculated using a standard solutions of sodium nitrite.

### 2.9. PGE2 Assay

PGE2 concentration was evaluated in cell culture supernatants obtained from RAW 264.7 cells treated with OLE (0.1 and 0.2 mg/mL) for 30 min before stimulation with Sodium Palmitate (SP) (0.5 mM) for 24 h. Prostaglandin E2 Enzyme Immunoassay (EIA) kit (Cayman Chemicals) was used according to the manufacturer’s instruction.

### 2.10. Flow cytometry

RAW 264.7 cells (5 × 10^5^ cells/well) or BMDMs (1 ×10^6^ cells/well) were plated into 6-well plates and allowed to adhere for 24 h. Then, cells were treated with OLE (0.1 and 0.2 mg/mL) for 30 min before stimulation with SP (0.5 mM) for 24 h. Aliquots of 5 × 105 cells were incubated with anti-Fc receptor (CD16/32) (Thermo Fisher Scientific) and then stained with the following murine monoclonal antibodies (mAbs): Phycoerythrin (PE)-Cyanine7-coniugated anti-CD206 (Thermo Fisher Scientific), Allophycocyanin (APC)-conjugated anti-arginase-1 (ARG1) (R&D System) and PE-Cyanine7-coniugated anti-NOS2 (Thermo Fisher Scientific). For intracellular staining of ARG1 and inducible nitric oxide synthase iNOS, cells were first fixed and permeabilized with Intracellular Fixation & Permeabilization Buffer (eBiosciences). Data were analyzed using BriCyte E6 (Mindray, Shenzhen, China). Dead cells were excluded by forward and side scatter characteristics. Statistics presented are based on 20,000 events.

### 2.11. Western Blot Analysis

RAW 264.7 cells (2 × 10^6^ cells/well) were plated into 6-well plates and allowed to adhere for 24 h. Then, cells were treated with OLE (0.1 and 0.2 mg/mL) for 30 min before stimulation with SP (0.5 mM) for additional 30 min (for nuclear and cytoplasmatic extracts) or 24 h (for total extracts). Whole-cell or nuclear extracts were prepared as previously described [23]. Protein concentration was measured by the Bradford method (Bio-Rad, Milan, Italy). 40 µg of proteins were separated by sodium dodecyl-sulfate polyacrylamide gel electrophoresis (SDS-PAGE) and transferred to nitrocellulose filter membranes using the Trans-Blot Turbo Transfer Starter System (Bio-Rad, Milan, Italy). Membranes were blocked with 5% low-fat milk in PBS with 0.1% Tween 20 (PBST) at room temperature for 2 h and, then, were incubated with the following primary antibodies: NF-κB p65 XP (#8242; Cell Signaling), IκB-α (#9242, Cell Signaling), NF-E2-related factor 2 (NRF2) (sc-722; Santa Cruz Biotechnology), KEAP1 (#4678; Cell Signaling), SOD2/MnSOD (ab13533; Abcam) iNOS (#39898; Cell Signaling), COX-2 (#4842; Cell Signaling), Glyceraldehyde 3-phosphate dehydrogenase (GAPDH) (#2118; Cell Signaling), α-TUBULIN (#3873; Cell Signaling), overnight at 4 °C. After 3 washes with PBST, the membranes were incubated with anti-mouse (Santa Cruz Biotechnology) or anti-rabbit (Jackson ImmunoResearch) secondary antibody, horseradish peroxidase (HRP) conjugate, for 2 h at room temperature. The membranes were developed with the ChemiDoc™ MP Imaging System (Bio-Rad, Milan, Italy) by the ECL chemiluminescence method. Band intensities were quantified using Image Lab Software and expressed as arbitrary units (A.U.).

### 2.12. MTT Assay

Cell viability was measured by 3-(4,3-(4,5-dimethylthiazol-2-yl)-2,5 diphenyltetrazolium bromide 5-dimethylthiazol-2-yl)-2, 5-diphenyltetrazolium bromide (MTT) assay. RAW 264.7 cells (0.5 × 10^5^ cells/well) were seeded on 96-well plates and treated with OLE (0.1 and 0.2 mg/mL) for 30 min before stimulation with SP (0.5 mM) for 24 h. Then, medium was removed and 200 µL of MTT (0.25 mg/mL) was added to each well. After 3 h incubation, the formazan crystals were solubilized in dimethyl sulfoxide (DMSO) (Merck KGaA, Darmstadt, Germany). Absorbance was measured by using a microplate spectrophotometer (MultiskanTM GO Microplate Spectrophotometer) at 490 nm.

### 2.13. Bone Marrow Derived Macrophages (BMDMs) Generation and Treatment

Bone marrow cells were obtained from femurs and tibias of C57BL/6 mice and cultured for 7 days in Roswell Park Memorial Institute (RPMI) 1640 medium containing 10% FBS, 10 mM HEPES buffer, penicillin (100 U/mL), streptomycin (100 µg/mL) and 25 ng/mL of mouse M-CSF at 37 °C in 5% CO_2_ atmosphere. Additional 5 mL of culture medium was added every second day. On the seventh day, BMDMs were harvested, plated at 1 × 10^6^ cells/well and treated with OLE (0.2 mg/mL) for 30 min before stimulation with SP (0.5 mM) for 24 h.

### 2.14. Statistical Analysis

Data are expressed as mean ± standard error of mean (SEM) (*n* ≥ 3). Statistical analyses were performed by using GraphPad Prism 6.0 software program (GraphPad Software, Inc., San Diego, CA, USA). One-way analysis of variance (ANOVA) with post-hoc Bonferroni’s test was performed to determine differences between groups. *p* < 0.05, *p* < 0.01 or *p* < 0.001 was considered statistically significant and indicated by *, ** or ***, respectively.

## 3. Results

### 3.1. Characterization of OLE Composition

The phenolic contents of OLE were calculated on the base of corresponding standard concentration–response curves obtained through HPLC-DAD analysis. The phenolic compounds identified belong to four main groups: oleuropeosides (oleuropein), flavonols (rutin), flavones (apigenin, apigenin-7-O-glucoside and luteolin), phenylethanoid (tyrosol and hydroxytyrosol). Oleuropein (174.64 mg/g) was detected at the highest concentration in OLE, followed by hydroxytyrosol (26.65 mg/g), tyrosol (0.64 mg/g) and apigenin-7-O-glucoside (0.43 mg/g). The other compounds analyzed were found only in trace amounts as shown in Table 1. Our data are in accordance with the findings reported by [24] who indicated oleuropein as a predominant phenolic compound, which represented more than 50% of the total phenolic compounds identified in alcoholic extract from olive leaves.

### 3.2. OLE Suppresses TNF-α, IL-6 and IL-1β Production in FFAs-Stimulated RAW 264.7 Macrophages

**Figure 1:**
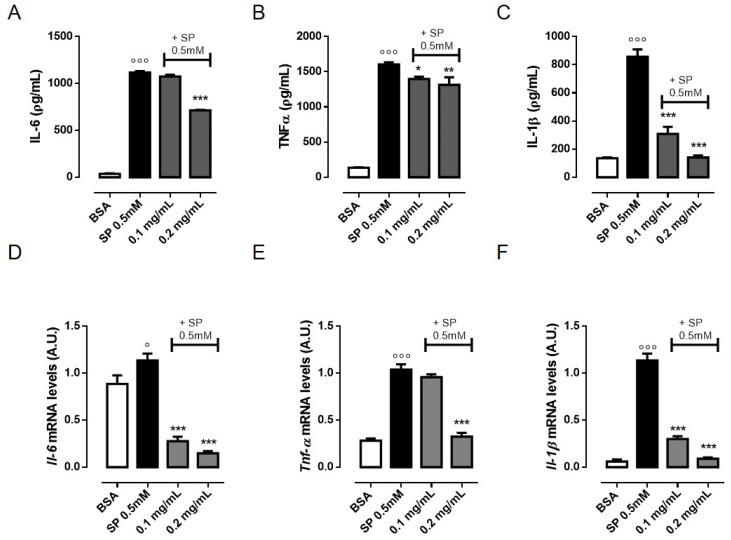
Olive Leaf Extract (OLE) reduces the production of pro-inflammatory cytokines in free fatty acids- (FFAs)-stimulated macrophages. RAW 264.7 cells were treated with OLE (0.1 and 0.2 mg/mL) for 30 min before stimulation with sodium palmitate (SP) 0.5 mM. (**A**–**C**) The levels of inflammatory cytokines tumor necrosis factor (TNF)-α, interleukin (IL)-6 and IL-1β were determined in the cell culture medium after 24 h by ELISA assay. (**D**–**F**) Relative mRNA levels of *Il-6, Tnf-α* and *Il-1β* in RAW 264.7 macrophages were determined by Real Time-PCR (RT-PCR) analysis after 6 h. Values are expressed as mean ± standard error of mean (SEM) from three independent experiments. ° *p* < 0.05, °°° *p* < 0.001 indicate significant effect of SP compared with vehicle-treated cells; * *p* < 0.05, ** *p* < 0.01, *** *p* < 0.001 indicate significant effect of OLE compared with SP-stimulated cells. BSA, bovin serum albumin.

Obesity-associated insulin resistance is associated with increased levels of pro-inflammatory cytokines, such as TNF-α, IL-1β, and IL-6 [25]. In obese adipose tissue, excess of saturated FFAs such as palmitate was reported to activate resident macrophages with production of pro-inflammatory cytokines that compromise insulin action [26]. To determine to what extent OLE modifies the FFAs-induced inflammatory response, we first studied the mRNA expression and the amount of TNF-α, IL-6 and IL-1β in RAW 264.7 cells exposed to 0.5 mM SP and pre-treated with OLE (0.1 and 0.2 mg/mL). The concentrations of SP used in the present study are in reference to the concentration of circulating free fatty acids in obesity [27]. The MTT assay revealed that SP 0.5 mM decreased cell viability whereas both concentrations of OLE, which was added 30′ prior to SP stimulation, did not significantly affect cell viability (Supplementary Material Figure S1). RAW 264.7 macrophages stimulated with SP showed increased level of pro-inflammatory cytokines in the culture supernatant as well as of their transcriptional levels compared to vehicle-treated cells. In contrast, OLE supplementation significantly diminished the secretion of all inflammatory mediators tested and markedly reduced their mRNA expression in a concentration-dependent manner (Figure 1A–F).

### 3.3. OLE Attenuates FFAs-Induced Oxidative Stress in RAW 264.7 Macrophages by Activating NRF2

**Figure 2:**
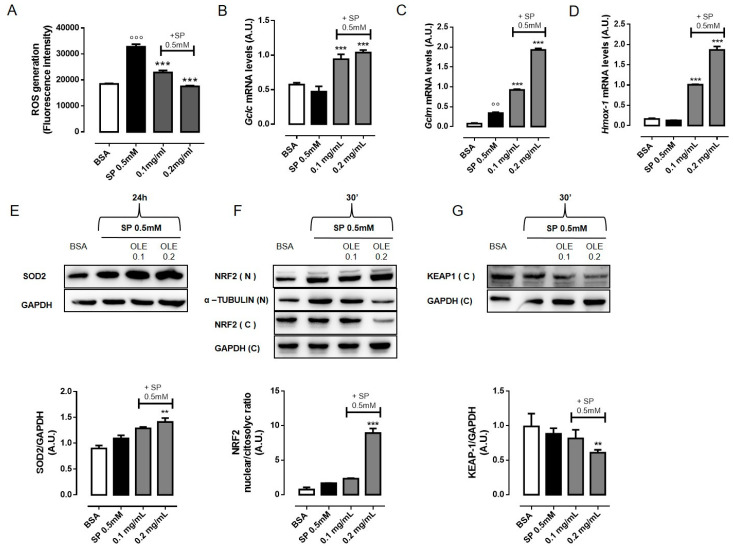
Olive Leaf Extract (OLE) attenuates free fatty acids (FFAs)-induced oxidative stress in macrophages by activating NF-E2-related factor 2 (NRF2) transcription factor. RAW 264.7 cells were treated with OLE (0.1 and 0.2 mg/mL) for 30 min before to stimulation with sodium palmitate (SP) 0.5 mM. (**A**) Intracellular reactive oxygen species (ROS) levels were measured using the fluoroprobe 2′,7′-dichlorofluorescein-diacetate (H2DCF-DA) after 24 h. (**B**–**D**) Relative mRNA levels of *Gclc*, *Gclm* and *Hmox-1* in RAW 264.7 macrophages were determined by Real time-PCR (RT-PCR) after 6 h. (**E**) Representative image of mitochondrial superoxide dismutase (SOD2) protein expression detected by western blot and relative densitometric quantification. (**F**) Representative images of NRF2 detected by western blot, in nuclear (N) and cytosolic (C) extract, and densitometric quantification of the nucleus/cytosol ratio. (**G**) Representative images of Kelch-like ECH-associated protein 1 (KEAP1) protein detected by western blot and densitometric quantification. Glyceraldehyde 3-phosphate dehydrogenase (GAPDH) and α–tubulin were used as an internal control. Values are express as mean ± standard error of mean (SEM) from three independent experiments. °° *p* < 0.01, °°° *p* < 0.001 indicate significant effect of SP compared with vehicle-treated cells; ** *p* < 0.01, *** *p* < 0.001 indicate significant effect of OLE compared with SP-stimulated cells.

Accumulating evidence suggests that oxidative stress is the leading cause of adipose tissue inflammation and of the pathogenesis of obesity-associated co-morbidities [28,29,30]. An important source of ROS in obesity is from ATMs. Recent reports have shown that certain saturated FFAs such as palmitate cause mitochondrial dysfunction and induce ROS production [26]. To investigate the effects of OLE on cell redox homeostasis, we measured intracellular ROS levels in SP-stimulated RAW 264.7 cells pre-treated with OLE (0.1 and 0.2 mg/mL) using the fluoroprobe H2DCFDA. Exposure of macrophages to SP 0.5 mM for 24 h significantly increased intracellular ROS generation whereas pre-treatment with OLE significantly decreased it in a concentration-dependent manner bringing back ROS to the basal level (Figure 2A). The oxidative homeostasis in normal cells is regulated by several anti-oxidant enzymes, such as superoxide dismutase (SOD), catalase (CAT) and glutathione peroxidase (GSX). To evaluate macrophages’ antioxidant defense capacity, we determined the expression levels of glutamate-cysteine ligase (GCL) and heme oxygenase-1 (HMOX-1) antioxidant enzymes. GCL, consisting of a catalytic (GCLC) and a modulatory (GCLM) subunits, is the rate-limiting enzyme in the glutathione (GSH) synthesis. In RAW 264.7 macrophages, OLE significantly enhanced gene expression of *Gclc*, *Gclm* and *Hmox-1* in a concentration-dependent manner (Figure 2B–D). Of interest is that increased expression of SOD can limit oxidative damage in obese mice [31]. Hence, we evaluated the protein expression of the mitochondrial SOD (SOD2). We found that SOD2 protein expression was significantly increased following OLE treatment in SP-stimulated RAW 264.7 macrophages (Figure 2E). These results suggest that OLE is able to regulate ROS production induced by SP stimulation in RAW 264.7 macrophages by promoting the expression of antioxidant proteins. Considering the indispensable role of the transcription factor NF-E2-related factor 2 (NRF2) in protecting cells from oxidative insults, we next sought to determine the involvement of NRF2 in OLE-antioxidant activity. We found that stimulation of RAW 264.7 macrophages with SP 0.5 mM promoted NRF2 protein migration into nuclei as a consequence of increased ROS level. Interestingly, OLE (0.2 mg/mL) pre-treatment significantly increased NRF2 nuclear translocation. NRF2 is predominantly degraded through the ubiquitination-mediated proteasome pathway. Under normal conditions, Kelch-like ECH-associated protein 1 (KEAP1) sequesters NRF2 into the cytosol and promotes its ubiquitination and proteasomal degradation; stimuli as ROS or electrophilic insults promote the dissociation of the NRF2-KEAP1 complex, and the subsequent NRF2 nuclear translocation [32]. Accordingly, we observed that KEAP1 protein level was significantly reduced in the cytosol of OLE treated cells (Figure 2F,G).

### 3.4. OLE Suppressed M1 Pro-Inflammatory Phenotype in FFAs-Stimulated RAW 264.7 Macrophages

**Figure 3:**
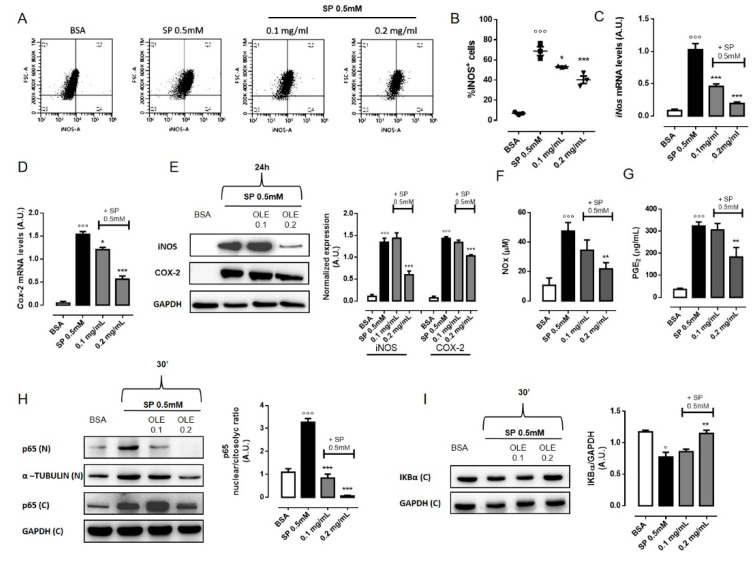
Olive Leaf Extract (OLE) suppresses M1 pro-inflammatory phenotype in free fatty acids (FFAs)-stimulated macrophages. RAW 264.7 cells were treated with OLE (0.1 and 0.2 mg/mL) for 30 min before stimulation with sodium palmitate (SP) 0.5 mM. (**A**) Representative plot and (**B**) relative quantitative analysis of intracellular inducible nitric oxide synthase (iNOS), expression in RAW 264.7 macrophages evaluated by flow cytometry after 24 h. Relative mRNA levels of *iNos* (**C**) and *Cox-2* (**D**) in RAW 264.7 macrophages were determined by Real Time-PCR (RT-PCR) after 24 h. (**E**) Representative image of iNOS and cyclooxigenase-2 (COX-2) proteins expression detected by western blot and relative densitometric quantification. Glyceraldehyde 3-phosphate dehydrogenase (GAPDH) was used as an internal control. (**F**) The levels of nitric oxide (NO) were measured in the cell culture medium after 24 h by the Greiss reaction. (**G**) The levels of prostaglandin E2 (PGE2) were measured in the cell culture medium after 24 h by the Enzyme Immunoassay (EIA). (**H**) Representative image of p65 detected by western blot, in nuclear (N) and cytosolic (**C**) extract, and densitometric quantification of the nucleus/cytosol ratio. (**I**) Representative image of IκB-α protein detected by western blot and relative densitometric quantification. α–TUBULIN and GAPDH were used as an internal control. Values are express as mean ± standard error of mean (SEM) from three independent experiments. ° *p* < 0.05, °°° *p* < 0.001 indicate significant effect of SP compared with vehicle-treated cells; * *p* < 0.05, ** *p* < 0.01, *** *p* < 0.001 indicate significant effect of OLE compared with SP-stimulated cells.

Macrophage M1/M2 status plays a fundamental role in the development of adipose tissue inflammation. In particular, obesity leads to a shift in ATMs phenotype from an anti-inflammatory M2-polarized state to an M1 pro-inflammatory state that supports insulin resistance [6]. M1 macrophages in obese adipose tissue express high levels of pro-inflammatory mediators, such as inducible nitric oxide synthase (iNOS), cytokines (IL-6, IL-1β and TNF-α) and reactive intermediates [33]. Thus, we next investigated whether OLE restrained FFAs-induced inflammation via regulation of macrophages polarization. As we have already showed above, OLE reduces the release of pro-inflammatory cytokines as well as ROS generation (Figure 1 and Figure 2). In addition, in order to detect M1 specific antigen, we performed the characterization of cell populations by flow cytometric analysis. Upon stimulation with SP 0.5 mM for 24 h, we found that the percentage of iNOS+ cells significantly increased from 6.9% to 78.8% compared to vehicle-treated cells. Interestingly, the pre-treatment with OLE 0.1 mg/mL and 0.2 mg/mL significantly decreased the percentage of iNOS+ cells to 53.4% and 40.3%, respectively (Figure 3A,B). Quantitative RT-PCR and western blot analysis also confirmed reduced expression of iNOS upon OLE treatment in SP-stimulated macrophages (Figure 3C,E). These finding were also confirmed in murine BMDMs (Supplementary Material Figure S2). Activation of M1 macrophages is normally accompanied also by increase of cyclooxigenase-2 (COX-2) expression. We found that *Cox-2* gene and protein were strongly upregulated in macrophages treated with SP. However, pre-treatment with OLE significantly and concentration-dependently reduced its expression (Figure 3D,E). In addition, the inhibitory effects of OLE on iNOS and COX-2 activity were confirmed by measuring their main products, NO and PGE2, respectively. Consistent with the previous results, we found that OLE significantly and concentration-dependently inhibited SP-induced NO (Figure 3F) and PGE2 (Figure 3G) production. We further examined the activation of NF-Κb, the key transcription factor that promotes the expression of pro-inflammatory genes in M1 cells [34]. NF-κB can be activated by the SFA palmitate, through the TLR4-mediated pro-inflammatory signaling pathway leading to M1 polarization status [26,35]. To explore the effect of OLE on the regulation of NF-κB signaling in SP-stimulated RAW 264.7 macrophages, we evaluated the nuclear translocation of NF-κB p65 subunit by western blot analysis. We found that upon stimulation with SP, p65 nuclear level were markedly increased whereas pre-treatment with OLE (0.1 and 0.2 mg/mL) significantly reduce p65 expression into the nuclei of macrophages in a concentration dependent-manner (Figure 3H). Additionally, we evaluated IκB-α protein expression, the inhibitor of NF-κB that normally retains NF-κB in the cytoplasm. We found that SP rapidly reduces IκB-α protein level in the cytosol while pre-treatment with OLE restores normal cytoplasmatic IκB-α level in RAW264.7 macrophages, suggesting that OLE inhibited SP-induced IκB-α degradation and, thus, NF-κB activation (Figure 3I).

### 3.5. OLE Promoted M2 Polarization in FFAs-Stimulated RAW264.7 Macrophages via PPARγ Activation

**Figure 4:**
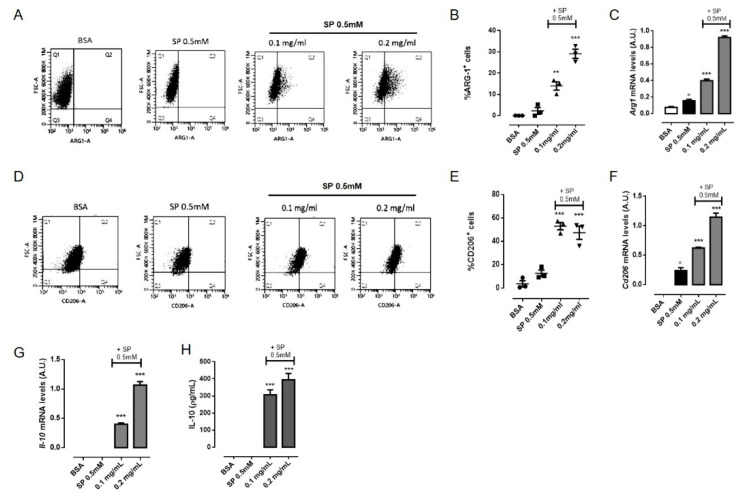
Olive Leaf Extract (OLE) promotes M2 polarization in free fatty acids (FFAs)-stimulated macrophages. RAW 264.7 cells were treated with OLE (0.1 and 0.2 mg/mL) for 30 min before to be stimulated with sodium palmitate (SP) 0.5 mM. (**A**) Representative plot and (**B**) relative quantitative analysis of intracellular arginase-1 (ARG-1) expression in RAW 264.7 macrophages evaluated by flow cytometry after 24 h. (**C**) Relative mRNA levels of *Arg-1* in RAW 264.7 macrophages were determined by Real Time-PCR (RT-PCR) after 24 h. (**D**) Representative plot and (**E**) relative quantitative analysis of mannose receptor C type 1 (CD206) expression in RAW 264.7 macrophages evaluated by flow cytometry after 24 h. (**F**,**G**) Relative mRNA levels of *Cd206* and *Il-10* mRNA in RAW 264.7 macrophages were determined by RT-PCR after 24 h and 6 h, respectively. (**H**) The levels of interleukin (IL)-10 cytokine were determined in the cell culture medium after 24 h by enzyme-linked immunosorbent assay (ELISA). Values are express as mean ± standard error of mean (SEM) from three independent experiments. ° *p* < 0.05 indicate significant effect of SP compared with vehicle-treated cells; ** *p* < 0.01, *** *p* < 0.001 indicate significant effect of OLE compared with SP-stimulated cells.

**Figure 5:**
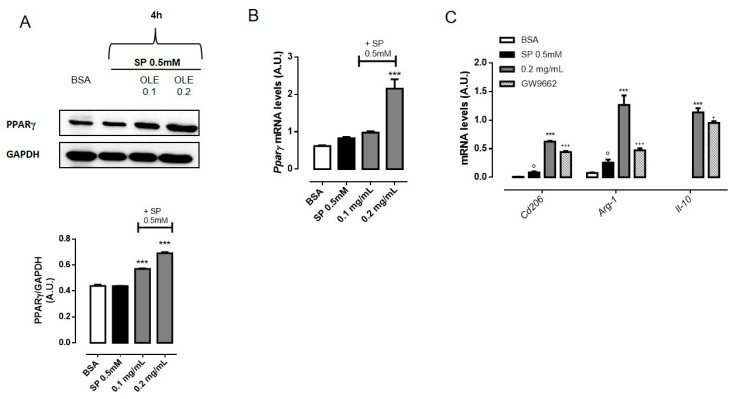
Olive Leaf Extract (OLE) activates peroxisome proliferator-activated receptor gamma (PPARγ) in free fatty acids (FFAs)-stimulated macrophages. RAW 264.7 cells were treated with OLE (0.1 and 0.2 mg/mL) for 30 min before stimulation with sodium palmitate (SP) 0.5 mM. (**A**) Representative images of PPARγ protein expression detected by western blot and relative densitometric quantification. Glyceraldehyde 3-phosphate dehydrogenase (GAPDH) was used as an internal control. (**B**) Relative mRNA levels of *Pparγ* were determined by Real Time-PCR (RT-PCR) after 4 h. (**C**) Relative mRNA levels of *Cd206, Arg-1* and *Il-10* in GW9662 pre-treated RAW 264.7 macrophages were determined by RT-PCR after 24 h. Values are express as mean ± standard error of mean (SEM) from three independent experiments. ° *p* < 0.05 indicate significant effect of SP compared with vehicle-treated cells; *** *p* < 0.001 indicate significant effect of OLE compared with SP-stimulated cells; + *p* < 0.05, +++ *p* < 0.001 indicate significant effect of GW9662 compared with OLE-treated cells.

As M2 macrophages ameliorate obesity-associated inflammation and insulin resistance [6], we evaluated whether OLE had any effects on macrophage polarization toward M2 phenotype. Alternatively activated macrophages found in lean adipose tissue express specific markers, including arginase-1 (ARG-1) and the mannose receptor C type 1 (CD206) and generate high levels of the anti-inflammatory cytokine IL-10, which plays a fundamental role in potentiating insulin sensitivity of adipocytes [33]. Thus, we characterized the phenotype of RAW 264.7 macrophages after treatment whit OLE by flow cytometric analysis. As shown in Figure 4, the percentage of ARG-1+ cells increased by approximately 30% 24 h post-treatment with OLE 0.2 mg/mL compare to vehicle- and SP-treated cells (Figure 4A,B). Similarly, the percentage of CD206+ cells increased by approximately 50% (Figure 4D,E). Quantitative RT-PCR also confirmed increased expression of *Arg-1* and *Cd206* mRNA upon OLE treatment (Figure 4C,F). Similar results were also confirmed in murine BMDMs (Supplementary Material Figure S2). Simultaneously, we found that SP treatment also slightly enhanced the expression of some M2 markers including *Arg-1* and *Cd206* mRNA. However, the significant elevation of the ratio of *iNOS* versus *Arg-1* or *CD206* in response to a high dose of SP is suggestive of M1 polarization (Supplementary Material Figure S2F). Furthermore, we determined the levels of IL-10 in cell culture supernatants of RAW264.7 macrophages as well as *Il-10* mRNA expression. As shown in Figure 4G,H, OLE treatment significantly increased IL-10 expression and production in a concentration-dependent manner. Several studies have defined a key role for PPARγ in controlling M2 macrophage polarization [36,37,38]. PPARγ is a ligand-inducible transcription factor that has been well documented to have anti-inflammatory effects in macrophages [39]. Thus, we further examined the activation of PPARγ in RAW 264.7 macrophages treated with OLE. Our data show that, OLE (0.2 mg/mL) significantly induce PPARγ expression in SP-stimulated macrophages (Figure 5A,B). Similar results were also confirmed in murine BMDMs (Supplementary Material Figure S2). Finally, to confirm the hypothesis that OLE regulates M1/M2 status by activating PPARγ, we used GW9662, a selective irreversible antagonist of PPARγ, to inhibit PPARγ transcriptional function. We found that GW9662 pre-treatment partially inhibited OLE-induced expression of M2 markers, such as *Cd206, Arg-1* and *Il-10* (Figure 5C) in SP-stimulated RAW 264.7 macrophages.

## 4. Discussion

Olive leaves represent a rich source of bioactive compounds with several beneficial effects for human health [20]. Previous studies reported the anti-inflammatory effect of oleuropein, the most abundant compound of the olive leaf extract, in lipopolysaccharide (LPS)-stimulated RAW 264.7 macrophages [40,41]. In addition, olive leaf constituents have demonstrated anti-obesity activity in terms of inhibition of adipogenic differentiation, reduction of body weight and improvement of lipid metabolism [42,43,44,45]. However, any of these studies evaluated the action of olive leaf extract on obesity-associated inflammation. It has been accepted that obesity coincides with a low-grade inflammatory state, referred as meta-inflammation, that mediates insulin resistance and it is closely related to the pathogenesis of obesity-associated diseases [2]. There are extensive evidences indicating that macrophages are primarily responsible for the inflammatory response into obese adipose tissue [4,5]. ATMs can polarize into two subtypes of activated macrophages: alternatively activated M2 macrophages that attenuate obesity-induced inflammation; and classically activated M1 macrophages that promote metabolic inflammation [6]. During weight gain macrophages undergo a ‘‘phenotypic switch’’ from an anti-inflammatory M2 phenotype to a pro-inflammatory M1 state, which contribute to insulin resistance [46]. In particular, a continuous and excessive exposure of ATMs to saturated FFAs, such as palmitate, promotes the secretion of pro-inflammatory mediators (cytokines, ROS and NO) that further supports M1 macrophages’ polarization and aggravate inflammation in the adipose tissue [47]. Thus, restoring the M2 phenotype might reduce meta-inflammation and protect from the risk to develop obesity-related diseases [15,16]. Several studies have demonstrated that some natural compounds have the potential to alleviate obesity-related inflammation via regulation of macrophage polarization [48,49,50]. In the current study, we confirm previously published results on the anti-inflammatory effect of oleuropein and showed, for the first time, the ability of OLE to induce a phenotypic switch of macrophage toward an M2-like phenotype in FFAs-stimulated macrophages. Prolonged treatment (24 h) of RAW 264.7 macrophages with high dose of SP (0.5 mM) was performed to mimic lipo-toxicity under obese condition. Consistent with many recently published studies [51,52] we found that 24 h treatment of macrophages with SP (0.5 mM) drove mouse macrophage polarization to a pro-inflammatory phenotype. Indeed, after being treated with SP, RAW 264.7 macrophages dramatically expressed several M1 markers; they exhibited increased production of ROS and inflammatory cytokines, TNF-α, IL-1β and IL-6, and showed increased expression of COX-2 and iNOS concomitant with the release of the respectively inflammatory mediators PGE2 and NO. Secretion of these factors supports adipose tissue inflammation and promotes insulin resistance throughout the activation of several inflammatory signal transduction pathways in macrophages and adipocytes [25]. Our data showed that pre-treatment with OLE significantly suppressed the releasing of the pro-inflammatory cytokines TNF-α, IL-1β and IL-6 in the supernatant of SP-activated RAW 264.7 cells with greater effect on IL-1β levels and mRNA. IL-1β is the major pro-inflammatory cytokine produced by ATMs and it is implicated in the development of obesity-associated insulin resistance [53,54]. Therefore, IL-1β might be a therapeutic target for the improvement of insulin sensitivity at tissue and systemic levels. In fact, a neutralizing antibody for IL-1β, Canakinumab, is currently under clinical trial for diabetes [55]. Obesity is also associated with increasing ROS levels, mainly produced by ATMs that contribute to induce insulin resistance and type 2 diabetes [29]. Indeed, exposure of RAW 264.7 cells to high concentration of SP increased ROS intracellular levels, while OLE had a remarkable suppressive effect on SP-induced ROS production. OLE-mediated suppression of oxidative stress was regulated by the transcription factor NRF2. NRF2−KEAP1 pathway is a key defense system for the protection of cells from oxidative stress [56]. To date, several natural and synthetic compounds have proven to be effective against obesity by inducing NRF2. In fact, NRF2 agonists demonstrated to reduce total body fat, plasma lipids levels, and to improve glucose tolerance and insulin resistance in HFD-fed mice [57,58]. In our experiments, we found that OLE also acts as NRF2 inducer. Indeed, it effectively reduced the abundance of KEAP1 in the cytosol and promoted the translocation of NRF2 into the nucleus of macrophages. Consequently, the activation of NRF2 correlated with the increased expression of its target antioxidant enzymes, such as HMOX-1, GCL and SOD2 responsible for ROS control in macrophages. An essential difference between M1-M2 polarization states is the metabolism of l-arginine that involves the enzymes ARG-1 and iNOS. In particular, iNOS produces reactive NO species with pro-inflammatory effects, while ARG-1 converts l-arginine to polyamines and collagen precursors that are crucial for tissue repair and remodeling [59]. In the present study, we found that OLE noticeably decreased iNOS expression and subsequent NO production induced from SP stimulation in RAW 264.7 macrophages. In addition, COX-2 and its mediator PGE2 were also significantly reduced by OLE. Concomitant with the reduction of M1 markers, RAW 264.7 macrophages treated with OLE exhibited enhanced expression of M2 specific markers including ARG-1 and CD206 as well as increased production of the anti-inflammatory cytokine IL-10. Taken together, these results demonstrated that OLE reduces M1 polarization and promote M2 remodeling of macrophages stimulated with high concentration of FFAs. The switch of macrophages from M1 to M2 phenotype was also examined by the expression of transcription factors that define macrophage profile and function. NF-κB is the key transcription factor for M1 pro-inflammatory macrophages, while PPARγ is considered as a master regulator of the M2 phenotype [35,38]. Several stimuli, such as ROS, TNF-α, LPS, IL-1β and SFAs, promote NF-κB activation and drive the expression of target genes [14]. Our data demonstrated that OLE markedly reduced the level of NF-κB p65 subunit into the nuclei of SP-stimulated RAW 264.7 while increasing the level of IκBα in the cytoplasm. Thus, OLE-mediated inhibition of NF-κB translocation and activation significantly contributed to the suppression of SP-induced inflammatory responses. PPARγ is a ligand-inducible transcription factor that regulates a variety of physiological processes, including glucose and lipid metabolism [60]. Moreover, PPARγ is implicated in the control of immunological events, mediating the differentiation and activation of immune cells to anti-inflammatory phenotypes [61]. Due to its role in macrophage polarization and regulation of inflammation, PPARγ has become an attractive pharmacological target for the development of drugs used for the treatment of metabolic disease in which activated macrophages play prominent pathogenic roles. Consequently, potent full agonists of PPARγ, thiazolidinediones, have been widely used for the treatment of type 2 diabetes in clinical practice [62]. Recently, it has been demonstrated that phenol fraction from virgin olive oil promotes M2 polarization in LPS-challenged human macrophages augmenting the transcriptional activity of PPARγ, which contributes to lower inflammation [63]. In addition, macrophage-specific deletion of PPARγ impairs M2 polarization and predisposes HFD-fed mice to develop obesity and insulin resistance [37]. Consistent with previous studies, we found that supplementation of OLE significantly increased the expression of PPARγ in SP-stimulated RAW 264.7 macrophages; further, inhibition of PPARγ with GW9662 significantly attenuated the ability of OLE to induce M2 macrophage polarization, suggesting that PPARγ is, at least in part, required for OLE to regulate macrophage phenotype. However, other studies observed that oleuropein or OLE inhibits PPARγ in adipocytes, which results in reduced adipogenesis and thermogenesis in vesical adipose tissue [42,64]. These contrary results might depend on the different cellular model used. Thus, oleuropein, or OLE, could be able to differently regulate PPARγ function based on the specific cell type examined. In conclusion, our study demonstrated that OLE regulates M1/M2 macrophage polarization in conditions of FFA-induced inflammation throughout two main actions: i) suppression of M1-mediate pro-inflammatory response by inhibiting NF-κB activity and up regulating NRF2-dependent genes; ii) enhancement of M2 polarization in a PPARγ-dependent manner. These finding indicate that polyphenols from olive leaves may be used as dietary supplementation for the prevention and treatment of obesity-associated inflammation and related comorbidities. However, further studies are required for a better understanding of the effects of OLE on the macrophages of the adipose tissue in diet-induced obese mice.

## Figures and Tables

**Table 1:** Olive Leaf Extract (OLE) polyphenolic characterization.

Phenolic Compound	Calibration Curve	Content in OLE (mg/g) *
Apigenin	Y = 4 × 10^7^x + 118566	0.01 ± 0.01
Apigenin-7-*O*-glucoside	Y = 5 × 10^7^x − 11131	0.43 ± 0.02
Hydroxy-tyrosol	Y = 650564x − 932.11	26.65 ± 0.08
Luteolin	Y = 6 × 10^7^x − 2632.8	0.36 ± 0.01
Oleuropein	Y = 3 × 10^6^x − 11515	174.64 ± 2.32
Rutin	Y = 2 × 10^7^x + 396.8	0.24 ± 0.02
Tyrosol	Y = 8 × 10^6^x − 4951.2	0.64 ± 0.08

* Results were expressed as mg/g DW ± SD (*n* = 3). DW: dry weight; SD: standard.

## Supplementary Materials

The following are available online at https://www.mdpi.com/2072-6643/12/12/3663/s1, Figure S1: Effect of sodium palmitate (SP) on macrophages viability; Figure S2: Olive Leaf Extract (OLE) drives murine bone marrow-derived macrophages (BMDMs) towards M2 polarization.
