# Supplementary material for: Olive Leaf Extract, from Olea europaea L., Reduces Palmitate-Induced Inflammation via Regulation of Murine Macrophages Polarization
Source: Nutrients. 2020 Nov 28;12(12):3663. doi: 10.3390/nu12123663 (PMC7761141; doi:10.3390/nu12123663)
Supplement: Supplementary file 1 [file nutrients-12-03663-s001.pdf]

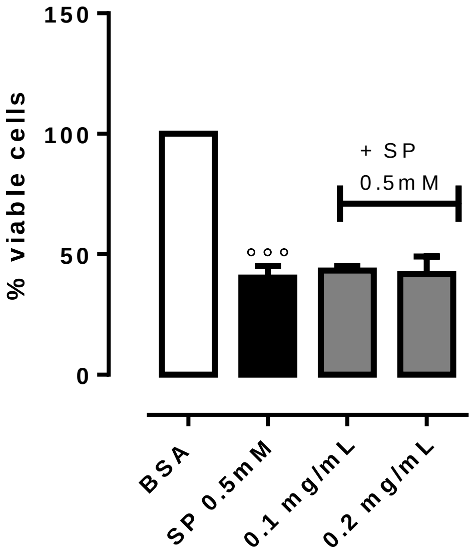

Figure S1. Effect of sodium palmitate (SP) on macrophages viability. The effect of SP (0.5mM) with or without Olive Leaf Extract (OLE) (0.1-0.2 mg/mL) on the viability of RAW 264.7 macrophages was measured by 3-(4,3-(4,5-dimethylthiazol-2-yl)-2,5 diphenyltetrazolium bromide 5-dimethylthiazol-2-yl)-2, 5-diphenyltetrazolium bromide (MTT) assay. Values are express as mean ± standard error of mean (SEM) from three independent experiments. °°° $p < 0.001$ indicate significant effect of SP compared with vehicle-treated cells.

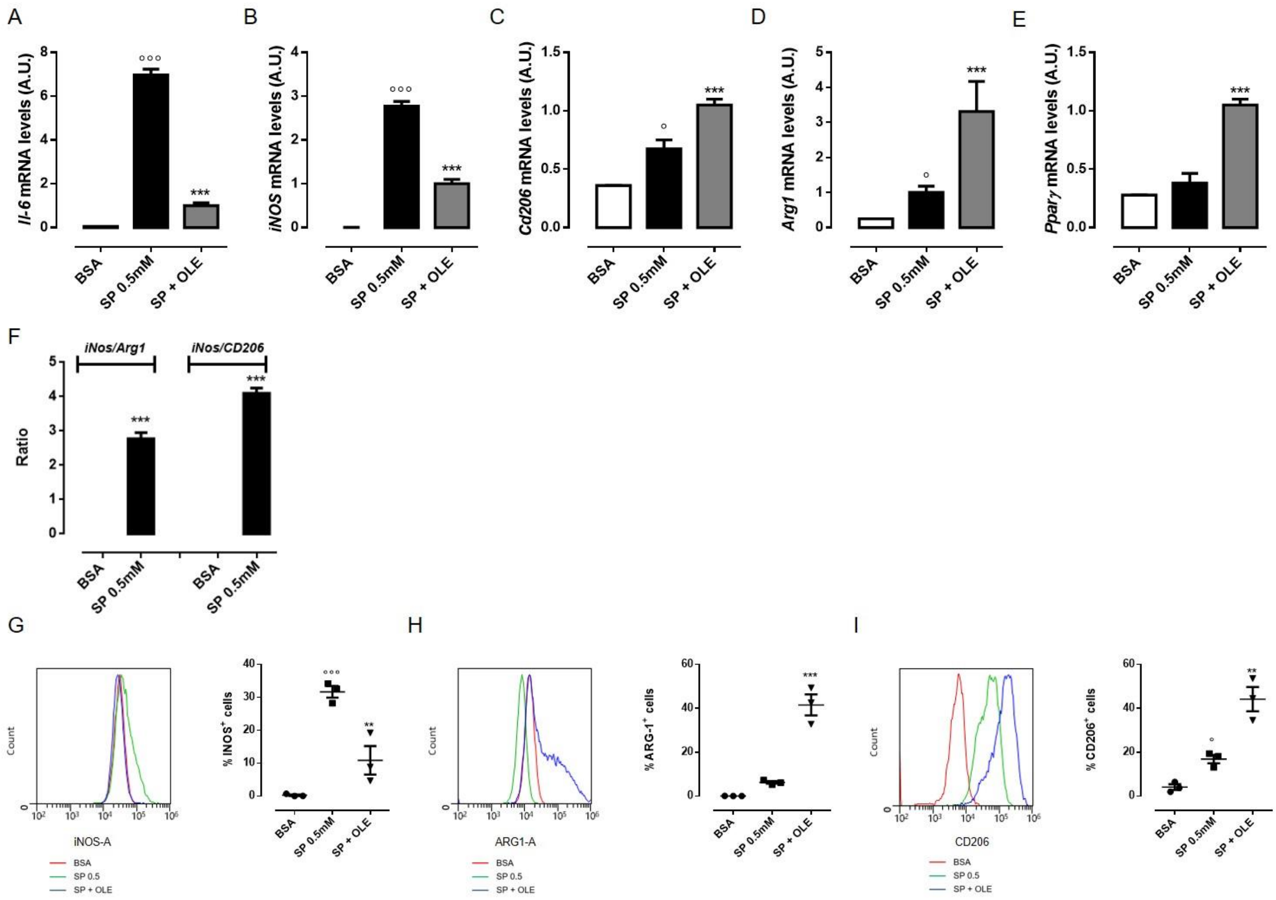

Figure S2. Olive Leaf Extract (OLE) drives murine bone marrow-derived macrophages (BMDMs) Table 2. polarization. Bone marrow cells were differentiated to macrophages for 7 days. On day 7th BMDMs were treated with OLE (0.2 mg/mL) for 30 min before to be stimulated with sodium palmitate (SP) 0.5 mM. (A-E) Relative mRNA levels of *interleukin (Il)-6, inducible nitric oxide synthase (iNos), mannose receptor C type 1 (CD206), arginase-1 (Arg-1)* and *peroxisome proliferator-activated receptor gamma (Pparγ)* were determined by Real Time-PCR (RT-PCR). (F) Ratio of mRNA levels of *iNos* versus *Arg-1* and *Cd206*. (G-I) Flow cytometric analysis of iNOS, ARG1 and CD206 expression and relative quantitative analysis in BMDMs after 24 h. Values are express as mean ± standard error of mean (SEM) from three independent experiments. ° $p < 0.05$, °°° $p < 0.001$ indicate significant effect of SP compared with vehicle-treated cells; ** $p < 0.01$, *** $p < 0.001$ indicate significant effect of OLE compared with SP-stimulated cells.
